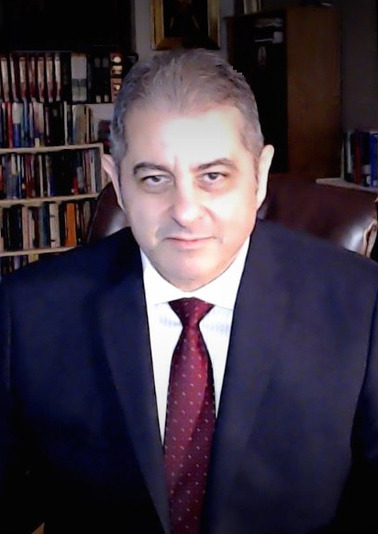# Exploring the Chemical Space of Mycobacterial Oxidative Phosphorylation Inhibitors Using Molecular Modeling

**DOI:** 10.1002/cmdc.202400303

**Published:** 2024-09-20

**Authors:** Islam K. Matar, Zhongmin Dong, Chérif F. Matta

**Affiliations:** ^1^ Department of Chemistry Saint Mary's University 923 Robie Street B3H 3C3 Halifax, NS Canada; ^2^ Department of Chemistry and Physics Mount Saint Vincent University 166 Bedford Highway B3M 2J6 Halifax, NS Canada; ^3^ Department of Biology Saint Mary's University 923 Robie Street B3H 3C3 Halifax, NS Canada

**Keywords:** Tuberculosis, Hansen's disease (leprosy), NTM pulmonary disease, mycobacterial oxidative phosphorylation, computational drug discovery

## Abstract

Mycobacteria are opportunistic intracellular pathogens that have plagued humans and other animals throughout history and still are today. They manipulate and hijack phagocytic cells of immune systems, enabling them to occupy this peculiar infection niche. Mycobacteria exploit a plethora of mechanisms to resist antimicrobials (e. g., waxy cell walls, efflux pumps, target modification, biofilms, etc.) thereby evolving into superbugs, such as extensively drug‐resistant tuberculosis (XDR TB) bacilli and the emerging pathogenic *Mycobacterium abscessus* complex. This review summarizes the mechanisms of action of some of the surging antimycobacterial strategies. Exploiting the fact that mycobacteria are obligate aerobes and the differences between their oxidative phosphorylation pathways *versus* their human counterpart opens a promising avenue for drug discovery. The polymorphism of respiratory complexes across mycobacterial pathogens imposes challenges on the repositioning of antimycobacterial agents to battle the rise in nontuberculous mycobacterial infections. *In silico* strategies exploiting mycobacterial respiratory machinery data to design novel therapeutic agents are touched upon. The potential druggability of mycobacterial respiratory elements is reviewed. Future research addressing the health challenges associated with mycobacterial pathogens is discussed.

## Graphical abstract

Intracellular pathogens are exceedingly clinically challenging, including pathogenic mycobacteria. Many global efforts are currently in action to battle these notorious disease agents. This review provides an overview of mycobacteria, along with their associated diseases, pathogenesis, and exploitable respiratory vulnerabilities to supplement contemporary antimycobacterial discovery.

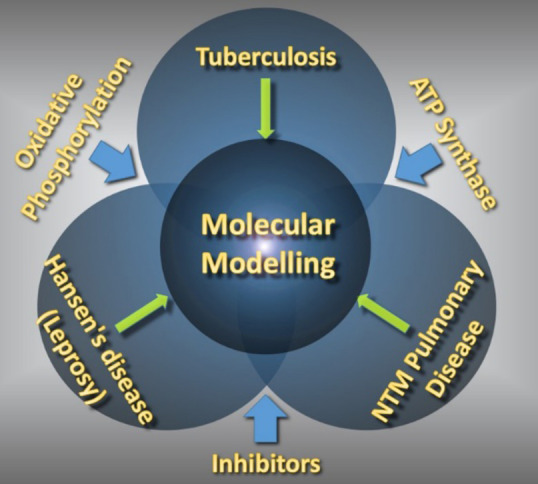

## 1 Introduction

Mycobacteria are abundant in the environment, and the majority of this genus is non‐pathogenic to healthy individuals under normal circumstances. Nonetheless, obligate pathogenic species are exceptionally malicious; epitomized by the notorious *Mycobacterium tuberculosis*.^[1]^ Mycobacteria are highly adaptable because of their resistant waxy cell walls, their ability to form biofilms,[^2^, ^3^] and being capable of surviving stressful conditions through remarkable dormancy systems.^[4]^ Moreover, pathogenic species are exceedingly cunning in manipulating and evading the immune systems of their hosts.

Diseases caused by mycobacteria can generally be categorized into three major categories; tuberculosis, leprosy, and nontuberculous mycobacterial infections.^[5]^ Nontuberculous mycobacteria (NTM) are opportunistic pathogens that tend to infect the lungs and soft tissues of several animal species, including humans.^[3]^ NTM infections have been notably more frequent in the last few decades, and they are highly resistant to most antibiotics.[^3^, ^6^, ^7^] Additionally, treatment of NTM infections usually has poor therapeutic outcomes because of the lengthy course of treatment and poor patient adherence.^[6]^ On the other hand, tuberculosis ranked as the second highest cause of death among single infectious agents globally, following COVID‐19, in 2022.^[8]^ It led to nearly double the number of deaths compared to HIV/AIDS.^[9]^ Lastly, leprosy is a disease that has been stigmatized historically, leading to social isolation of affected individuals.^[10]^ The route of transmission of leprosy is not completely worked‐out yet, but it is thought to spread *via* respiratory droplets from infected individuals.^[11]^ The hallmark of leprosy is nerve damage, leading to sensory and motor impairment, which often results in deformities and disabilities.[^10^, ^11^] Therapeutic innovations are, therefore, imperative to conquer the ancient yet persistent mycobacterial morbidity.

Targeting the energy metabolism of mycobacteria has been established as an effective strategy for treating drug‐resistant mycobacterial infections.[^12^, ^13^, ^14^, ^15^, ^16^] The oxidative phosphorylation pathway is a targetable vulnerability for those organisms because they are generally obligate aerobes,[^12^, ^15^, ^16^] with few exceptional adaptations mentioned in the literature.[^17^, ^18^] Strategies for targeting the oxidative phosphorylation pathway include diminishing the respiratory proton‐motive force through uncoupling agents,^[14]^ inhibiting the electron transport chain (ETC) complexes,^[15]^ and obstructing ATP synthase.^[16]^

The mechanistics of mycobacterial respiratory complexes (ETC complexes and ATP synthase collectively) are not yet fully understood. Recent studies provided insights into the structure and function of these complexes,[^19^, ^20^, ^21^, ^22^, ^23^, ^24^] while others have explored the supercomplex assembling interactions between them,[^25^, ^26^] particularly in the context of drug development. These studies have identified potential drug targets and structural combinations, shedding light on the complex interplay of the mycobacterial respirasome. Howbeit, further research is needed to fully elucidate their intricate mechanistics.^[27]^

Molecular modeling, and more broadly computational chemistry, is a valuable tool for deciphering the mechanistic details of such biological processes, on both the molecular and electronic scales, and designing efficacious chemical entities to modulate them.[^28^, ^29^, ^30^, ^31^, ^32^, ^33^] Ergo, implementing those computational techniques to the understanding of mycobacterial oxidative phosphorylation may beneficially contribute to further advancement of antimycobacterial drug discovery and, thereby, supplement the global efforts in this evolutionary battle.

## 2 Mycobacteria: A Persistent Global Health Burden

### 2.1 Overview

Mycobacteria are a group of aerobic, non‐motile, and rod‐shaped bacteria that belong to the family *Mycobacteriaceae*. They are characterized by their unique cell wall structure, which contains mycolic acids, giving them resistance to many chemical treatments, including the Gram stain. Instead, mycobacteria are often classified as acid‐fast bacteria because they retain the Ziehl‐Neelsen stain even after acid‐alcohol treatment. There are more than 190 recognized species within the *Mycobacterium* genus,^[34]^ which stands as the sole genus within the *Mycobacteriaceae* family.^[34]^ Mycobacteria display considerable diversity with regard to their capacity to induce diseases in humans. Certain mycobacterial species are categorized as strict pathogens, while others function as opportunistic pathogens or are considered non‐pathogenic.[^1^, ^35^, ^36^]

The discovery of mycobacteria is historically attributed to the German physician, microbiologist, and the 1905 Nobel Laureate in Physiology or Medicine, Robert Koch. In 1882, Koch identified and described *Mycobacterium tuberculosis*, the bacterium responsible for causing tuberculosis (TB).[^37^, ^38^] Nevertheless, the history of mycobacteria is hypothesized to extend to the Jurassic period, nearly 150 million years ago.[^38^, ^39^] According to literature, vertebral lesions characteristic of tuberculosis have been discovered in mummies dating back to the pre‐Columbian era in Peru and the pre‐dynastic period in Egypt.[^38^, ^40^, ^41^] Moreover, leprosy (Hansen's disease) has records that can be traced back to around 600 BC; which are believed to have originated from India. Those records are considered to be the oldest reliable source describing structural manifestations that match the current medical diagnostic criteria for leprosy. The social stigma associated with leprosy has led to uncertainties in detecting its chronological and geographical origins.^[42]^

### 2.2 Mycobacterial Taxonomy

Mycobacteria belong to the *Mycobacterium* genus which holds over 190 species^[34]^ and falls within the *Mycobacteriaceae* family.[^34^, ^43^] The genus is categorized under the *Mycobacteriales* order, *Actinomycetes* class, *Actinomycetota* phylum, and *Bacteria* domain (https://lpsn.dsmz.de/genus/mycobacterium).^[43]^ The genus was initially introduced in 1896 to accommodate organisms that were then perceived as intermediates between bacteria and fungi.^[44]^ Mycobacteria can be classified using various methods, taking into consideration factors such as their clinical significance, growth characteristics, and genetic properties.

In terms of clinical significance, mycobacteria can be classified based on their impact on human health. Some mycobacterial species are evident vicious pathogens of humans, as well as animals, including the *Mycobacterium tuberculosis* complex (MTC)[^1^, ^45^, ^46^] and the *Mycobacterium leprae* complex (MLC).[^1^, ^47^, ^48^] The MTC comprises eleven species,[^45^, ^49^, ^50^] seven of which have been reported in cases of human tuberculosis, namely *M. tuberculosis*,[^45^, ^49^, ^50^, ^51^, ^52^] *M. bovis*,[^45^, ^50^, ^51^, ^52^] *M. africanum*,[^45^, ^49^, ^50^, ^51^, ^52^] *M. microti*,[^45^, ^51^, ^53^, ^54^, ^55^] *M. canettii*,[^45^, ^51^, ^56^, ^57^, ^58^] *M. caprae*,[^59^, ^60^, ^61^, ^62^, ^63^] and *M. orygis*.[^64^, ^65^, ^66^, ^67^, ^68^, ^69^] The primary causative agent of human tuberculosis is *M. tuberculosis*.[^35^, ^37^, ^38^, ^45^, ^51^, ^70^, ^71^] On the other hand, the MLC contains two species which are the etiologic agents of human leprosy; *M. leprae* and *M. lepromatosis*.[^72^, ^73^] Finally, nontuberculous mycobacteria (NTM), mycobacteria other than those which inflict tuberculosis and leprosy,[^1^, ^74^] constitute the majority of the *Mycobacterium* genus and have been viewed classically as seldom pathogenic environmental organisms. However, NTM are currently recognized as opportunistic pathogens with notable clinical relevance.^[74]^ The *Mycobacterium avium* complex (MAC) is considered the most prevalent and clinically significant pathogen within the NTM group.[^1^, ^6^, ^75^] MAC is the leading cause of NTM pulmonary disease, particularly in individuals with underlying respiratory ailments, e. g., bronchiectasis and chronic obstructive pulmonary disease (COPD).[^1^, ^6^, ^75^, ^76^, ^77^] Originally, only two species were hosted in the MAC, *Mycobacterium avium* and *Mycobacterium intracellulare*. Presently, the complex encompasses twelve species,[^76^, ^78^, ^79^] including the two initial ones. The names of the additional species are *Mycobacterium arosiense*, *M. bouchedurhonense*, *M. chimaera*, *M. colombiense*, *M. lepraemurium*, *M. marseillense*, *M. paraintracellulare*, *M. timonense*, *M. vulneris*, and *M. yongonense*.

In reference to growth characteristics, mycobacteria are traditionally categorized by comparing their *in vitro* growth rates and pigmentation.[^80^, ^81^] This classification system was established in 1959 by the esteemed American microbiologist and physician Ernest Runyon,^[80]^ hence it is known as the Runyon classification system. According to this system, Mycobacteria are broadly divided into either slow‐growing or rapid‐growing species. The slow‐growing branch is subdivided into three groups; Group (Runyon) I, II, and III. On the other hand, the rapid‐growing species are all assigned to one group, Group IV. Group I are labeled Photochromogens because they exhibit a range of yellow–red pigments upon growth in light. Group II, termed Scotochromogens, are similarly pigmented as the first group with the distinction of pigment production regardless of light exposure. Group III do not display significant pigmentation and thus are identified as Nonchromogens.

**Figure 1:**
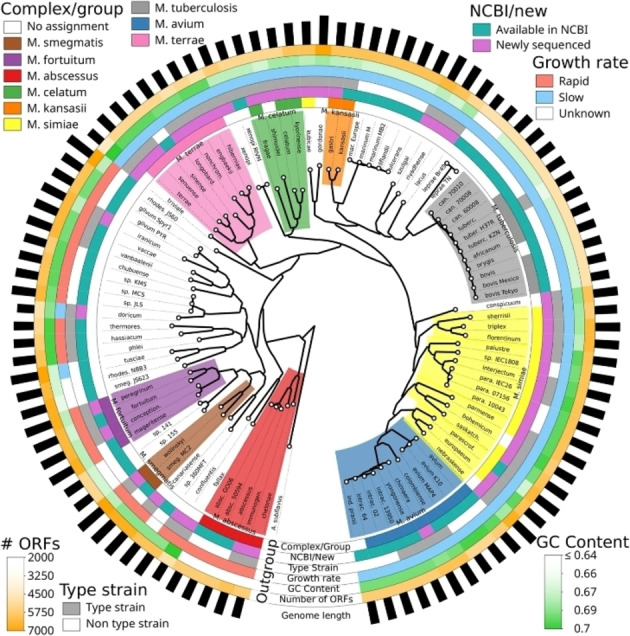
Circular dendrogram depicting a whole‐genome phylogenetic tree of the Mycobacterium genus. Growth rate is illustrated among other annotations for each species. (Reproduced from Ref. [83], **©** 2017 Creative Commons by 4.0).

As for genetic properties, mycobacteria can be extensively segregated on the basis of their molecular phylogeny. Cultivating mycobacteria in vitro under axenic culture conditions is generally more challenging compared to other bacteria and in some cases, particularly the MLC, is still unachievable at all.[^4^, ^72^, ^73^, ^82^] Hence, advancements in molecular phylogenetic technologies had a profound impact on mycobacterial taxonomy and practically led to the reconstitution of the genus in recent years.[^34^, ^83^] Currently, comparative genomic studies are the standard for the taxonomic classification of mycobacteria,[^34^, ^72^, ^73^, ^83^, ^84^, ^85^, ^86^, ^87^, ^88^] as well as other microorganisms. Nonetheless, the conventional Runyon growth rate terminology, i. e. slow/rapid growing species, still carries on in contemporary literature[^83^, ^86^, ^87^, ^88^] (Figure 1).

### 2.3 Mycobacterial Diseases

Mycobacterial diseases, entrenched in the annals of human history, have long captured the fascination of researchers and the collective concern of healthcare communities worldwide. These diseases, often characterized by their enigmatic nature and the resilience of their causative agents, Mycobacteria, have left an indelible mark on the trajectory of public health. Here, we will explore the landscape of the three major diseases inflicted by mycobacterial pathogens: Tuberculosis, leprosy, and NTM pulmonary disease.

#### 2.3.1 Tuberculosis

Historically, tuberculosis (TB) was commonly referred to as “consumption” due to the progressive weight loss and wasting away of individuals with the disease. TB has been unfathomably notorious across all of human history that it was denominated “*The Captain of All the Men of Death*” by John Bunyan in the seventeenth century.[^37^, ^89^, ^90^, ^91^] This “*Captain of Death*” has claimed the lives of an estimated 1.3 million people in 2022,^[92]^ with no prospective early retirement. Approximately 10.6 million TB incidences were reported in 2022;^[92]^ nearly one case every three seconds. The global economic burden of TB control is 13 billion dollars per annum.^[92]^. Global strategies and efforts are in motion to terminate this TB epidemic by the end of this decade, 2030.[^8^, ^93^, ^94^] This global health objective constitutes an essential element of Target 3.3 of the United Nations’ Sustainable Development Goal 3.[^8^, ^93^, ^94^, ^95^]

##### 2.3.1.1 Pathology

TB is caused by the *Mycobacterium tuberculosis* complex, with the *Mycobacterium tuberculosis* (Mtb) species leading the majority of human infections. Besides its deleterious effects on lungs, TB may as well harm other organs, e. g., the kidneys and central nervous system. TB spreads through the air when an infected person coughs or sneezes, releasing respiratory droplets containing the bacteria. TB can present in two forms: latent TB infection and active TB disease. In latent TB infection, the bacteria are present in the body but are not causing symptoms, and the person is not contagious, yet running the risk of conversion into an active TB case at later times. In active TB disease, the bacteria are actively multiplying, causing symptoms, and the person is contagious. Common symptoms of active pulmonary TB include a persistent cough, chest pain, weight loss, fatigue, fever, night sweats, and coughing up blood.[^70^, ^71^, ^96^, ^97^]

Upon initial exposure to tuberculosis bacilli, primary tuberculosis manifests as localized infection in the central segment of the lungs, known as the Ghon focus. In many cases, the Ghon focus transitions into a quiescent phase, termed latent tuberculosis, which can be reactivated upon immunosuppression or re‐infection. Primary progressive tuberculosis, a subset of cases, occurs shortly after first exposure and is prevalent in vulnerable populations. On the other hand, secondary tuberculosis typically develops after a prolonged period of latency, often years after the initial infection, and results from the reactivation of latent tuberculosis. The lesions in secondary tuberculosis, located in the lung apices, usually involve cavitation, distinguishing it from primary progressive tuberculosis, in which cavitation or tissue damage is less conspicuous. Pulmonary granuloma, a conglomerate of immune cells engulfing the tuberculosis bacilli, of characteristic caseous necrosis and multinucleated giant cells, is the hallmark of TB.^[98]^

Mtb is a diabolical parasite with a formidable evolutionary history that enabled it to thrive in such a sophisticated infection niche. Among the extraordinary qualities contributing to the evolutionary success of Mtb is its ability to hijack an essential component of its host's immune system, the alveolar macrophage.[^70^, ^96^, ^98^, ^99^, ^100^, ^101^] Macrophages play a pivotal role in both facets of the immune system, innate and adaptive immunity, starting with the digestive phagocytosis of pathogens all the way to antigen presentation to T lymphocytes. Macrophages must be able to successfully phagocytose and digest a pathogen before further initiation of an immune response.^[100]^ This digestive phagocytic process involves several stages, initiating by the formation of an early phagosome upon pathogen engulfment.^[102]^ Then, maturation of the early phagosome to a late phagosome is achieved through the integration and activation of vacuolar ATPase (V‐ATPase) proton pumps, causing acidification of the phagosome's lumen, which is followed by a series of fusion events with late endosomes.[^102^, ^103^] Next, the late phagosome fuses with the lysosomal compartment to create a phagolysosome capable of pathogen digestion.[^102^, ^103^] Finally, the process is terminated upon resolution of the phagosome to maintain the functionality of the macrophage.[^102^, ^104^] Mtb uses secretory phosphatases, in addition to other mechanisms, to interfere with early phagosome maturation, consequently blocking later steps that lead to the biogenesis of the phagolysosome.[^100^, ^101^, ^105^, ^106^, ^107^] Mtb protein‐tyrosine phosphatase A (PtpA) hinders the incorporation of the V‐ATPase into the membrane of the early phagosome,[^100^, ^101^, ^105^, ^106^] thereby subverting the hallmark of phagosome maturation, the phagosome acidification. Additionally, Mtb PtpA inhibits the phagosome‐lysosome fusion by dephosphorylating the human vacuolar protein sorting 33B (VPS33B).[^100^, ^101^, ^105^] Furthermore, Mtb secreted acid phosphatase M (SapM) impedes the fusion between the early phagosome and the late endosomes via the continuous dephosphorylation of phosphatidylinositol 3‐phosphate (PI3P) on the membrane of the early phagosome.[^100^, ^101^, ^105^, ^106^, ^107^] The arrest of the phagosome maturation allows Mtb to dwell and reproduce inside the alveolar macrophage, inaugurating the infection.

Mtb is well recognized as a facultative intracellular pathogen, referring to its preference to reside and replicate within phagocytic cells, particularly macrophages.[^101^, ^106^, ^108^] This intracellular lifestyle deems it necessary to sustain the viability of the infected cells while the pathogen replicates.^[109]^ Meanwhile, it is also in favor of the pathogen to spread the infection to other cells through bursting out of the infected phagocytes.^[109]^ Ergo, Mtb has been demonstrated to dispatch both anti‐apoptotic and pro‐necrotic signaling molecules along the path of its infection, enabling it to optimally exploit its host environment.[^100^, ^101^, ^106^, ^109^, ^110^, ^111^] Among these signaling molecules, protein‐tyrosine phosphatase B (PtpB) was reported to reduce the activity of caspase‐3, by promoting the phosphorylation of Akt, thereby downregulating apoptosis.^[105]^ Besides, Mtb also stifles the apoptotic signals of tumor necrosis factor (TNF) by secreting NuoG which detoxifies the reactive oxygen species produced by macrophages, as well as neutrophils.[^100^, ^106^, ^109^] Conversely, Mtb instigates necrosis in macrophages through the destructive actions of the ESAT‐6 protein,[^106^, ^112^, ^113^] phthiocerol dimycocerosates,[^106^, ^113^, ^114^] and outer membrane channel protein CpnT.[^106^, ^115^]

To add insult to injury, Mtb is also capable of escaping the phagosome and the phagolysosome and translocating to the cytosol.[^106^, ^114^, ^116^, ^117^] This is accomplished via damaging the membrane of the phagosome using its phthiocerol dimycocerosates and the phagolysosome using the ESAT‐6 protein.[^106^, ^113^, ^114^, ^116^] In the cytosol, Mtb forms structures similar to biofilms called “cords” which contribute to its pathogenicity.[^106^, ^118^] In response, the alveolar macrophage resorts to autophagy in an attempt to control the bacterial load in the cytoplasm.[^100^, ^101^, ^106^, ^111^, ^114^, ^117^, ^119^, ^120^] Mtb then counters with virulence factors, like PE_PGRS proteins, which can lead to arresting the host's autophagy attempts.[^106^, ^120^] It is a battle for survival, and the outcomes are in major determined by the host's immune system fitness.

##### 2.3.1.2 Treatment

Tuberculosis is treated using combination therapy of antibiotics[^8^, ^121^] to ensure the eradication of the infection. The duration of TB therapeutic regimens ranges between 4–9 months, depending on the medical status of the patient and the results from the antimicrobial susceptibility tests.[^8^, ^121^] Common drugs used in TB therapeutic regimens include rifamycin derivatives (rifampicin and rifapentine), isoniazid, ethambutol, pyrazinamide, and streptomycin.[^8^, ^121^] Multidrug resistance in TB therapy remains a hurdle in the path of ending its epidemic.[^8^, ^92^, ^93^, ^94^]

#### 2.3.2 Leprosy

From the outset, a clarification suggested by one of the anonymous reviewers of this paper is pertinent. The disease in this discussion has no simple relation to biblical leprosy, as leprosy within historical and, especially, biblical contexts referred to a number of dermatological conditions generally associated with white spots or scaling which does not necessarily meet the diagnostic criteria for Hansen's disease, that is, medical leprosy. Etymologically, the word “leprosy” stems from the Greek word *λέπρα* (*lepra*) which was translated from the Hebrew word *צָרַעַת* (*zara'at*), which is not in sync with the known historical fact that Hansen's disease was brought to the Middle East with Alexander the Great's armies upon its return from India *ca*. 325–324 BCE, much later than the latest writings of the Old Testament.[^122^, ^123^] The mycobacterial disease discussed in this section will be addressed, henceforth, as “Hansen's disease” for scholarly precision and to avoid contributing to the existing stigma.^[124]^ Hansen's disease is a chronic infection that mainly targets the skin and peripheral nervous system. The disease may progress to cause disabilities in severe cases that receive no medical attention.^[125]^ There were 174,087 new incidences reported in 2022, with the majority of the cases belonging to Africa and South‐East Asia.^[126]^ One of the World Health Organization (WHO)’s global strategies aims to eradicate the disease by 2030.^[127]^

##### 2.3.2.1 Pathology

Hansen's disease is caused by the *Mycobacterium leprae* complex; *Mycobacterium leprae* and *Mycobacterium lepromatosis* species.[^48^, ^82^, ^128^] The transmission mechanism of the disease remains unclear,[^48^, ^82^, ^125^, ^129^, ^130^] however, inhalation of respiratory droplets containing the bacteria for a prolonged period (several months) is the proposed main route of transmission.[^48^, ^125^, ^130^] The presentation and severity of the disease are majorly dependent on the nature of the immune response exhibited by the infected individual.^[131]^ The T helper (Th) 1 and Th2 paradigm, particularly, is considered pivotal in determining the disease prognosis;[^131^, ^132^, ^133^, ^134^] which is typically described on the basis of two extreme poles namely tuberculoid and lepromatous Hansen's disease.[^48^, ^82^, ^131^, ^133^, ^134^] In the tuberculoid pole, a strong cell‐mediated immune response is displayed which is characterized by a shift in the differentiation of T helper cells towards the Th1 form. The Th1 polarized response is associated with the secretion of cellular immunity cytokines, including Interferon‐gamma and Interleukin‐2, which activate cytotoxic T cells, macrophages, and natural killer cells leading to a more significant control of the infection in comparison to the lepromatous form.[^131^, ^132^, ^133^, ^134^] Therefore, tuberculoid cases present with few well‐demarcated skin lesions characterized by granuloma formation and low bacillary count, hence referred to as paucibacillary cases.[^48^, ^82^, ^131^, ^133^] On the contrary, the lepromatous extreme displays a weak cell‐mediated immune response due to Th2 polarization, which is accompanied by the release of humoral immunity cytokines, including Interleukin‐4 and Interleukin‐5, resulting in the activation of B cells and the production of antibodies.[^131^, ^132^, ^133^, ^134^] This type of immune response is ineffective because of the intracellular nature of the infection, ergo, the infection disseminates to other tissues and organs of the body.[^131^, ^132^, ^133^, ^134^] Consequently, lepromatous cases present with numerous ill‐marked and elevated skin lesions all over the body. The histopathology of lepromatous skin lesions is characterized by foam cells and high bacillary count; multibacillary.[^48^, ^82^, ^131^, ^133^] The Ridley‐Jopling classification system defines those two poles along with an intermediary spectrum of the clinical features of Hansen's disease (HD).[^48^, ^82^, ^133^, ^135^]

The species of the *Mycobacterium leprae* complex are obligate intracellular pathogens with a notable proclivity for macrophages and Schwann cells.[^131^, ^132^, ^136^] The HD bacilli possess a characteristic virulence factor located within their cell walls known as phenolic glycolipid‐1 (PGL‐1). During the infection of Schwann cells, the HD bacilli use their PGL‐1 to bind to the laminin‐2 external protein of the Schwann cells, triggering a complex cascade that eventually leads to the internalization of the pathogen.[^82^, ^137^, ^138^] The infection of the Schwann cells, predominantly occurring in peripheral nerves, results in the demyelination of the affected nerves subsequently impeding the conduction of nerve signals and ultimately causing paresthesia and loss of motor function.[^48^, ^131^] The HD bacteria also employ their PGL‐1 to infect macrophages through binding to the complement component 3 expressed at the surface of the macrophage.[^131^, ^138^] Within the macrophage, the HD bacilli, similar to the tuberculosis bacilli, translocate from the phagolysosome to the cytosol where it utilizes the nutritional resources of the host cell to survive and replicate.^[116]^ It is understood that the HD bacilli resist the destructive mechanisms of the phagosome before translocating from the phagolysosome to the cytosol in phagocytic cells, which happens approximately 2 days after the infection.^[116]^ However, the literature on the molecular mechanisms describing the influence of HD bacilli, specifically, on phagosome maturation during the pre‐translocation phase is rather sparse compared to other mycobacterial pathogens, such as *Mycobacterium tuberculosis* and *Mycobacterium abscessus*, and is mostly inferred on the basis of similarity between the mechanistics of mycobacterial infections.^[139]^ On the other hand, the focus in the literature has shifted to the role of autophagy in the containment of HD bacilli perforated phagosomes and cytosolic HD bacilli, both mediated by the membrane‐destructive effects of the ESAT‐6 protein.[^134^, ^136^, ^139^, ^140^, ^141^] The formation of autolysosomes, in the case of cytosolic bacilli, and autophagolysosomes, in the case of bacilli perforated phagosomes, through successful autophagy is currently considered a decisive factor in the prognosis of the disease.[^136^, ^141^]

The significance of innate immunity elements in Hansen's disease has notably changed over recent years. The differential polarization of macrophages into pro‐inflammatory (M1) and anti‐inflammatory (M2) phenotypes, in particular, is becoming more recognized as a pillar in the immunity against Hansen's disease.[^131^, ^132^, ^134^, ^136^, ^140^] Paucibacillary cases have been reported to exhibit a predominant M1 macrophage population, while M2 macrophages were more prevalent in the multibacillary ones.[^131^, ^132^, ^134^, ^136^, ^140^] The role of the Jagged 1 (JAG1) protein in mediating the interferon‐gamma stimulation of endothelial cells towards M1 macrophage differential polarization has also been recently identified.^[142]^ In addition, studies have demonstrated a prevalence of dendritic cells in tuberculoid skin lesions, compared to lepromatous lesions, suggesting their importance in immunostimulation and granuloma formation in Hansen's disease.[^143^, ^144^, ^145^] The differential expression of the cluster of differentiation 1 (CD1) proteins, responsible for presenting lipid antigens to T cells, between the two polar forms of Hansen's disease indicates the role of dendritic cells in the activation of the adaptive immunity against the HD bacilli.[^144^, ^146^] Additionally, an upregulation of keratinocytes displaying Intercellular Adhesion Molecule 1 (ICAM‐1) was observed in tuberculoid HD.^[147]^ However, the role of keratinocytes in HD immunity remains ambiguous, because stimulating their proliferation, using recombinant granulocyte‐macrophage colony‐stimulating factor (GM‐CSF), didn't reduce the bacillary count.^[148]^ Finally, pentraxin‐3 (PTX3) released by neutrophils was proposed as a biomarker for distinguishing type 2 reactions, erythema nodosum leprosum (ENL), from type 1 reactions, reversal reaction.^[140]^ Type 1 and type 2 reactions are immune‐mediated complications that can occur in non‐tuberculoid HD patients.^[48]^

Overall, the pathology of Hansen's disease requires further investigation. The complex nature of its host‐pathogen interactions renders the molecular pathology of the disease a tangled puzzle that is yet to be deciphered. Hansen's disease continues to be an excellent model for immunology studies, deeming it necessary to acquire a deeper understanding of this important disease.

##### 2.3.2.2 Treatment

Hansen's disease is currently treated using a multi‐drug therapeutic regimen comprising dapsone, rifampicin, and clofazimine. The therapeutic duration varies depending on the bacillary load in skin lesions, 6 months for paucibacillary cases and 12 months for multibacillary ones.^[125]^ Regular monitoring and management of immune‐mediated complications are also crucial to ensure therapeutic success and prevent disabilities.^[48]^

#### 2.3.3 NTM Pulmonary Disease

NTM infections are a rising health problem, especially NTM lung disease. A systematic review in 2022 indicated that 82.1 % of global studies on NTM pulmonary infections denote an exacerbation in the prevalence of those infections, with a 4.0 % annual increase in the number of cases per thousand individuals.^[149]^ Members of the *Mycobacterium avium* complex and *Mycobacterium abscessus* complex are the top NTM pulmonary pathogens, with the former dominating all NTM infections.[^149^, ^150^] The molecular identification of the causative species is crucial for ensuring therapeutic success and proper patient education.^[151]^ Furthermore, the anonymity in NTM infections has been a peculiar frustration for healthcare practitioners and patients altogether, to the extent that a patient committed suicide in the past in response to this medical oblivion.^[80]^ Populations at risk of NTM pulmonary infections include geriatric (older than 65) persons, postmenopausal females, immunocompromised patients, and patients suffering from preexisting pulmonary conditions such as bronchiectasis, cystic fibrosis, and chronic obstructive pulmonary disease.^[152]^ Patients who have been previously infected with an NTM pulmonary pathogen continue to be vulnerable to being reinfected with NTM pulmonary pathogens, whether the same species or a different one, even after a successful therapeutic cure.^[151]^ For the scope of this review, pulmonary infections caused by the *Mycobacterium avium* complex (MAC) will be discussed.

##### 2.3.3.1 Pathology

MAC bacilli are abundant in environmental settings such as soil and water, and it is generally accepted that their entry into the human body occurs via respiratory inhalation.^[152]^ Upon entering the respiratory system, these pathogens adhere to the mucosal epithelial cells within the respiratory tract, subsequently infecting the macrophages tasked with their eradication, under normal conditions.[^150^, ^153^] The pulmonary macrophages uptake the invasive bacilli in an attempt to employ their regular defensive phagocytic mechanism, which relies on the successful formation of a phagolysosome. However, MAC possesses distinctive antigenic lipids known as glycopeptolipids (GPLs) in their cell walls. These GPLs impede the macrophages’ destructive processes by arresting the phagosome maturation pathway, thus enabling the bacteria's survival and proliferation within the pulmonary macrophages. The intracellular multiplication of these mycobacteria instigates an inflammatory response, drawing additional macrophages to the infection site.[^7^, ^150^, ^153^, ^154^] The newly arrived macrophages attempt to contain the infection through the formation of granulomas, which, as they enlarge, develop into nodules. This progression culminates in nodular bronchiectasis, wherein chronic inflammation inflicts damage on the bronchi, leading to scarring and dilation. Further, the impaired bronchi exhibit reduced mucociliary clearance and local immune function, diminishing their capacity to clear foreign materials from the lungs. Consequently, mucus accumulates in these dilated regions, creating an ideal breeding ground for bacterial proliferation. Nodular bronchiectasis typically evolves gradually, but in certain instances, a more rapid and severe form, known as fibrocavitary disease, emerges. In this condition, the mycobacteria invade lung tissue, causing extensive damage, severe fibrosis, and cavity formation, akin to tuberculosis. Given that MAC species are aerobic, these cavities predominantly form in the oxygen‐rich upper lobes of the lungs.[^155^, ^156^, ^157^] In exceptional cases, particularly among individuals with compromised immune systems, the infected macrophages can migrate to the lymphatics, disseminating the bacteria to other body parts, notably the spleen, liver, and bone marrow, and potentially spreading to the bloodstream, leading to disseminated MAC disease.^[158]^ Pulmonary MAC infections manifest symptoms including cough, fever, fatigue, weight loss, night sweats, shortness of breath, and recurrent respiratory infections. Disseminated MAC infections, on the other hand, may present with nonspecific symptoms such as fever, sweats, fatigue, and weight loss, accompanied by physical examination findings of hepatomegaly, jaundice, and lymphadenopathy.[^157^, ^159^, ^160^]

The interactions between the MAC bacilli and macrophages are dynamic, complex, and not yet fully understood.^[7]^ The reported interactions, so far, involve the manipulation of macrophage apoptosis, autophagy, and the arrest of phagosome maturation.[^7^, ^158^, ^161^] A very interesting study in 2011 used microscopy data to propose a model where MAC exploits macrophage apoptosis to infect other macrophages thereby spreading the intracellular infection. In the model, bacteria undergo phagocytosis by macrophages, followed by the induction of apoptosis in these immune cells a few days subsequent. During the apoptotic process, a portion of the bacteria is eradicated, whereas others manage to evade the apoptotic bodies, transitioning into the extracellular environment. Here, they are susceptible to efficient phagocytosis by a subsequent macrophage, potentially initiating a recurrent cycle. Concurrently, a different subset of bacteria remains within the apoptotic bodies. When these bodies are uptaken by new macrophages, some bacteria succeed in infecting these fresh host cells, while others are eliminated.^[158]^ Subsequent studies attempted to provide further explanation of the interplay between MAC and macrophage apoptosis utilizing MAC biofilms, different MAC subspecies, and MAC antigens.[^162^, ^163^, ^164^, ^165^, ^166^, ^167^, ^168^] Despite the insights provided by the aforementioned model and the ensuing literature, the conundrum of whether macrophage apoptosis thwarts or enables the MAC infection persists. On the other hand, GPLs expressed by MAC, precisely serovar 4‐specific glycopeptidolipid, were asserted to halt the phagosome‐lysosome fusion pathway.[^169^, ^170^] Finally, the literature on the role of autophagy in MAC infections is fairly limited, compared to *Mycobacterium tuberculosis* and *Mycobacterium leprae*, with some authors underscoring the protective role of autophagy in MAC infections. Yet, it remains unclear if MAC is capable of hijacking or perverting its host's autophagy mechanism.^[161]^

##### 2.3.3.2 Treatment

The recommended treatment protocol for MAC pulmonary infections involves a multidrug regimen, typically consisting of a macrolide, usually azithromycin, combined with rifampin and ethambutol. The use of a macrolide is essential, as it serves as a cornerstone in MAC therapy due to its bactericidal and immunomodulatory properties. Rifampin, a potent antimycobacterial agent, complements the macrolide, while ethambutol adds further efficacy by inhibiting bacterial cell wall synthesis. The duration of treatment is protracted, often extending for at least 12 months after culture conversion to negative, to mitigate the risk of relapse. The management of MAC pulmonary infections necessitates individualized approaches, considering factors such as drug susceptibility patterns, the extent of disease, and the patient's overall health. Regular monitoring of treatment response through sputum cultures and radiological assessments is imperative to guide therapeutic adjustments and ensure optimal clinical outcomes. In nodular‐bronchiectatic patients, the multidrug dose is provided three times per week. In aggressive bronchiectatic, fibrocavitary, or macrolide‐resistant cases, parenteral streptomycin or amikacin is added to the original drug combination, and the dosing frequency is switched to a daily dose. Amikacin liposome inhalation is combined with the aforementioned four‐drug regimen upon the persistence of positive sputum cultures after six months of therapy.^[151]^

### 2.4 Therapeutic Challenges and a Dire Necessity for Innovation

Treatment of mycobacterial infections is challenging due to several reasons including the lengthy therapeutic regimens involving multiple antimicrobial agents, side effects and poor patient adherence,^[171]^ antimicrobial resistance and persistence,[^172^, ^173^, ^174^, ^175^, ^176^, ^177^, ^178^, ^179^] host immunomodulation, and concomitant health complications (comorbidities and potential disabilities). These challenges necessitate the discovery and development of innovative therapeutic entities, and approaches, with improved potency, safety, and patient compliance. Strategies and areas of research to address these challenges include the identification and validation of novel drug targets;[^180^, ^181^, ^182^, ^183^, ^184^] host‐directed therapies;[^185^, ^186^, ^187^, ^188^] drug combination therapies;[^189^, ^190^, ^191^] advanced drug delivery technologies;^[192]^ and vaccines.^[193]^

Among the currently rousing antimycobacterial target spaces is the oxidative phosphorylation pathway, representing an invaluable vulnerability of those relentless pathogens.[^12^, ^15^, ^16^, ^181^, ^194^, ^195^] The next section will discuss the mycobacterial oxidative phosphorylation pathway from the perspective of drug discovery.

## 3 Mycobacterial Oxidative Phosphorylation: A Bioenergetic Vulnerability

### 3.1 Overview

Mycobacterial energy metabolism is a complex process that comprises two major pathways, substrate‐level phosphorylation and oxidative phosphorylation. Oxidative phosphorylation refers to the process by which cells of the majority of living organisms generate adenosine triphosphate (ATP), the cellular energy currency, through the transfer of electrons along the respiratory chain and the coupling of this electron transport to the phosphorylation of adenosine diphosphate (ADP) to form ATP. In mycobacteria, oxidative phosphorylation takes place along their inner bacterial membrane, which faces the cytoplasm on the inner side and the periplasm on the outer side. The process involves a series of protein complexes, commonly referred to as the respiratory chain complexes or the electron transport chain, that are embedded in the membrane. These complexes facilitate the transfer of electrons through a series of redox reactions, typically ending with oxygen as the final electron acceptor. The movement of electrons through these complexes is coupled to the pumping of protons across the membrane to the periplasmic space, creating an electrochemical gradient. This proton gradient is then utilized by the ATP synthase complex to drive the synthesis of ATP from ADP and inorganic phosphate. The electrochemical gradient, also termed proton motive force, is additionally used to fuel the movement of a plethora of chemical species across the membrane including polypeptides, antibiotics, and ions.[^12^, ^195^, ^196^, ^197^]

Because mycobacteria are generally obligate aerobes, these microorganisms rely upon oxidative phosphorylation for the production of the preponderance of their ATP requirements, under normal conditions.^[197]^ Howbeit, due to the convoluted intracellular nature of mycobacterial infections, which involve hypoxic stress upon entrapment in a phagosome, an autophagosome, or possibly a granuloma, the dependence of different species of mycobacteria on oxidative phosphorylation appears to vary. This could be observed upon inhibiting the ATP synthase of *Mycobacterium tuberculosis*, *Mycobacterium abscessus*, and *Mycobacterium avium* using bedaquiline, which results in a bactericidal effect in the former and a bacteriostatic effect in the latter two.[^198^, ^199^, ^200^] Ergo, it can be speculated that nontuberculous mycobacteria are less dependent on oxidative phosphorylation compared to *Mycobacterium tuberculosis* for survival, not growth. Other studies of ATP synthase inhibition using bedaquiline on miscellaneous NTM species of variable growth rates, other than the two aforementioned species, demonstrated bactericidal effects in some NTM species and bacteriostatic in others.[^201^, ^202^, ^203^, ^204^] Therefore, it is not clear whether this dissimilitude in bedaquiline susceptibility among different, non‐mutant, NTM species is a consequence of resistance to bedaquiline or variability in oxidative phosphorylation dependence for survival, provided their relative genetic similarity. Finally, taking into consideration the lack of sufficient genetic studies on the ATP synthases of NTM, other than *Mycobacterium smegmatis*, the extent of NTM species’ dependence on oxidative phosphorylation for survival remains an open question. Contrarily, and in the nature of *Mycobacterium tuberculosis*, bedaquiline exhibits a bactericidal effect on *Mycobacterium leprae*, indicating the indispensability of oxidative phosphorylation for its replicating and non‐replicating bacilli altogether.^[205]^

The essential nature of the oxidative phosphorylation pathway in mycobacteria, combined with the invaluable distinctions between the mycobacterial and mitochondrial respirasomes and ATP synthases, later discussed, remarkably accentuate the druggability of this antimycobacterial target space.[^12^, ^195^, ^196^, ^197^] Several inhibitors of mycobacterial oxidative phosphorylation have been proposed, some were submitted to the antimycobacterial discovery pipeline, and a few are currently in clinical trials including bedaquiline in phase three, and TBAJ‐587 in phase one.^[206]^ Since the major focus in the antimycobacterial discovery and development pipeline is *Mycobacterium tuberculosis*, therefore, the preponderance of the knowledge on mycobacterial oxidative phosphorylation is driven by the same perspective. Hence, the following parts of this section will discuss mycobacterial oxidative phosphorylation from the point of view of *Mycobacterium tuberculosis*.

### 3.2 Mycobacterial Oxidative Phosphorylation Metabolic Network

**Figure 2:**
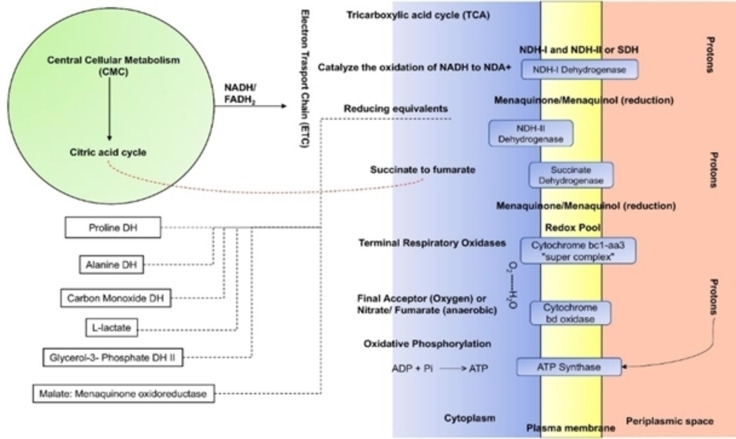
Schematic diagram illustrating the metabolic flexibility of the mycobacterial respiratory network. The left side of the diagram outlines the different sources supplying the electron transport chain with electron donors. Secondary electron donors’ feed by auxiliary dehydrogenases is sketched in black dotted lines. The right side depicts the organization and mechanism of the main respiratory pathway. The bidirectional connection between the central metabolism and the respiratory chain, mediated by succinate dehydrogenase, is shown as a red dotted line. (Reproduced with permission, Ref. [197], **©** 2022 Elsevier).

The mycobacterial oxidative phosphorylation metabolic network comprises fourteen enzymes, six of which are directly involved in the pathway, and the others support the pathway indirectly under stressful environmental conditions (Figure 2). This ingenious metabolic versatility elucidates the astounding adaptability of mycobacteria under the harsh intracellular conditions of phagocytic cells.

The six main enzymes of the pathway encompass three dehydrogenases; NADH dehydrogenase type I, NADH dehydrogenase type II, and succinate dehydrogenase I/II; two terminal oxidases; cytochrome bc1‐aa3 and cytochrome bd oxidase; and the ATP synthase. The additional secondary enzymes include five dehydrogenases of five substrates; Proline, alanine, glycerol‐3‐phosphate, L‐lactate, and carbon monoxide; two reductases of two substrates; fumarate and nitrate; and the malate: menaquinone oxidoreductase.^[197]^ Here, we will focus on the main enzymes of the pathway, analyzing their structural features and specific functions, along with examples of their known inhibitors.

### 3.3 Mycobacterial Oxidative Phosphorylation Main Enzymes

#### 3.3.1 NADH Dehydrogenase Type I

**Figure 3:**
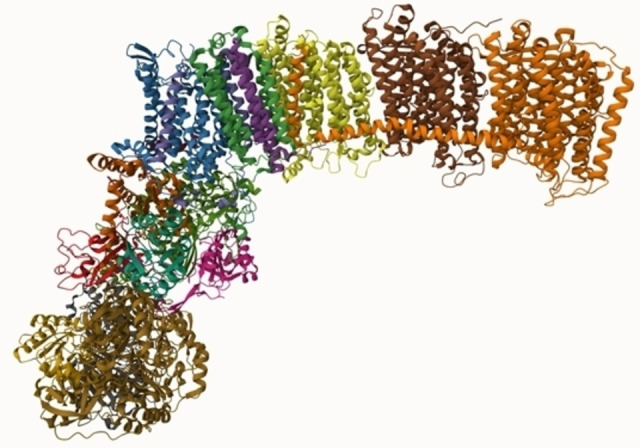
A representation of the mycobacterial NADH dehydrogenase type I, PDB ID: 8E9H, displaying its core subunits, where each subunit is colored differently. The top helical domain traverses the mycobacterial plasma membrane, while the bottom portion is solubilized in the cytoplasm. The figure was generated using Mol* Viewer.

Mycobacterial NADH dehydrogenase type I (NDH‐1) is a large, L‐shaped, transmembrane enzyme complex comprising 14 core subunits (Figure 3). The core subunits are encoded by the nuo‐operon, and the subunits are termed NuoA to NuoN respectively. The primary function of NDH‐1 is to catalyze the transfer of electrons from the reduced nicotinamide adenine dinucleotide (NADH), supplied mainly by the citric acid cycle and the oxidation of fatty acids, to either menaquinone or ubiquinone. Within the complex, there are several electron carriers which facilitate the transfer of electrons from NADH to the terminal quinone molecule. The initial electron carrier that receives the electrons from NADH is flavin mononucleotide, which then passes the electrons to continue their path along the complex through a series of 8 iron‐sulfur clusters till they are finally captured by the terminal quinone. Concurrent with the electron transfer, the enzyme contributes to maintaining the proton motive force by pumping 4 protons per each molecule of NADH.[^19^, ^197^]

The metabolic flexibility of mycobacteria enables them to survive in the absence of NDH‐1, therefore, the enzyme is considered a low‐value drug target. Examples of NDH‐1 inhibitors are rotenone, piericidinet, and pyridaben.^[197]^

#### 3.3.2 NADH Dehydrogenase Type II

Unlike NDH‐1, the NADH dehydrogenase type II (NDH‐2) of mycobacteria is a simpler monomeric isozyme, anchored to the cytoplasmic periphery of the membrane. The two forms of the enzyme are the products of the Ndh and NdhA genes. Similar to NDH‐1, the main function of NDH‐2 is to transfer electrons from NADH to either of the previously mentioned quinone species. This is, however, uncoupled with proton translocation, hence, the enzyme has no contribution to the proton motive force. NDH‐2 incorporates a flavin adenine dinucleotide (FAD) as its primary electron carrier. The enzyme was reported to have two quinone binding sites and does not contribute to the production of reactive oxygen species upon its oxidation of NADH, which provokes the host's immunity. Consequently, the mycobacterial cell favors the oxidation of NADH using NDH‐2.[^195^, ^197^, ^207^]

The Ndh isoform of NDH‐2 was demonstrated to be essential for growth and survival in mycobacteria according to mutation studies. Additionally, the eukaryotic electron transport chain does not include this prokaryotic protein. These premises support the druggability of NDH‐2, and consequently, many classes of NDH‐2 inhibitors have been published. The most important classes are phenothiazines and riminophenazines, which contain the two important drugs thioridazine and clofazimine, respectively.[^197^, ^208^]

#### 3.3.3 Succinate Dehydrogenase

**Figure 4:**
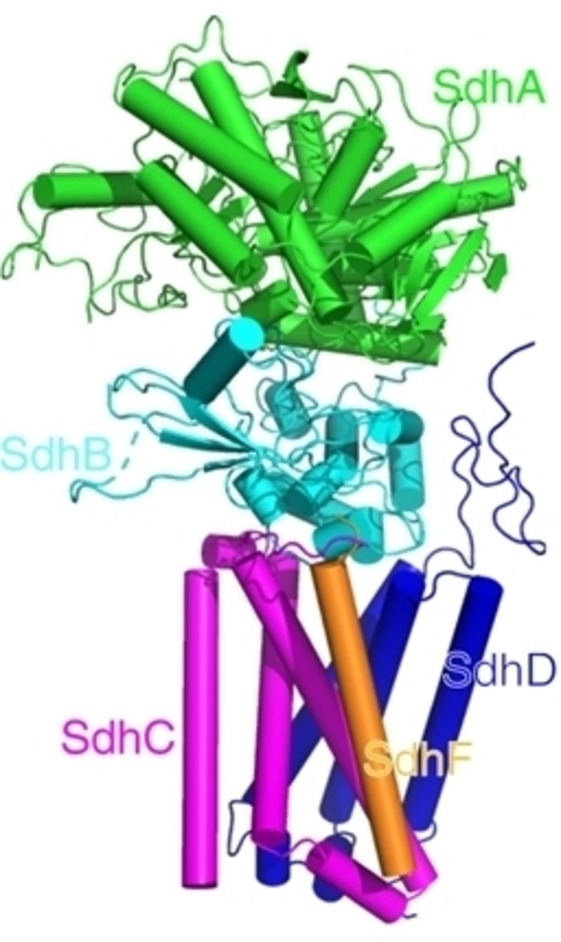
A display of the structure of mycobacterial succinate dehydrogenase 2 exhibiting the traditional four subunits, SdhA to SdhD, in addition to the recently discovered SdhF subunit. The membrane domain of the enzyme comprises subunits C, D, and F. (Reproduced with permission from Ref. [20], **©** 2020 Creative Commons by 4.0).

Mycobacteria have two distinct isoforms of succinate dehydrogenase (SDH), SDH1 and SDH2. Both isoforms are multimeric, SDH1 is trimeric while SDH2 has recently been reported to be pentameric (Figure 4).

SDH participates in both the citric acid cycle as well as the electron transport chain, connecting oxidative phosphorylation with the central metabolism. The enzyme catalyzes the oxidation of succinate to fumarate, which is coupled with the reduction of menaquinone in the electron transport chain, consequently referred to as succinate‐menaquinone oxidoreductase. The reverse process is catalyzed by the paralogous fumarate reductase enzyme in oxygen‐deficient environments. Both SDH isoforms have two major structural domains, a larger cytoplasmic domain and a smaller membrane domain. The cytoplasmic domain comprises two subunits, subunit A which includes a domain for the covalent binding of FAD along with another domain for substrate binding, and subunit B which encompasses three iron‐sulfur clusters that enable electron transfer from FAD to the membrane domain. The membrane domain of SDH2 is composed of subunit C and subunit D, while SDH1 has only subunit C to anchor it to the plasma membrane. The membrane domain, regardless of the difference in the number of subunits between the two isoforms, receives the electrons from the cytoplasmic domain and reduces the menaquinone to menaquinol.[^20^, ^21^, ^197^]

Mutation studies illuminated the fundamentalness of the SDH1 isoform for the adaptation and survival of mycobacteria by regulating the respiratory rate and replenishing the menaquinone pool. The essential role of SDH1 combined with its structural dissimilarity to its mitochondrial counterpart make it a potential drug target. Nevertheless, only a few inhibitors of SDH have been reported to date, among which are clofazimine, thioridazine, and triclosan.[^195^, ^197^]

#### 3.3.4 Cytochrome bc_1_‐aa_3_ Supercomplex

**Figure 5:**
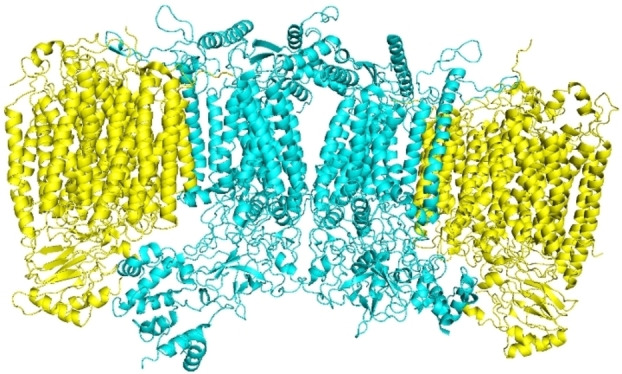
A representation of the mycobacterial cytochrome bc1‐aa3 supercomplex, PDB ID: 6HWH, where cytochrome bc1 (cyan) is sandwiched between cytochrome aa3 (yellow) molecules. Other co‐crystallized peptides and prosthetic groups are hidden for clarity purposes. The picture was produced using PyMOL.

This large mycobacterial respiratory supercomplex is the result of a dimeric assembly of one cytochrome bc1 sandwiched between two cytochrome aa3 molecules (Figure 5). The qcrCAB operon codes for the three subunits of cytochrome bc1; QcrA, QcrB, and QcrC. On the other hand, the ctaBCDE operon expression results in the four subunits of cytochrome aa3; CtaB, CtaC, CtaD, and CtaE. The primary function of this respiratory supercomplex is to oxidize menaquinol and shuttle the electrons to their final acceptor, oxygen. This process is accompanied by proton pumping to the periplasmic compartment of the mycobacterial cell, thus promoting the proton motive force. Both cytochromes of the assembly embrace an array of electron carrier species to catalyze the electron transfer from the reduced substrate to the recipient oxygen. In cytochrome bc1, the QcrA subunit holds an iron‐sulfur cluster, the QcrB subunit has two heme groups, and the QcrC is bound to an additional heme moiety. In cytochrome aa3, the CtaC subunit comprises two copper ions (Cu_A_), while the main catalytic CtaD subunit incorporates two heme ligands along with one copper ion (Cu_B_).[^25^, ^26^, ^195^, ^197^, ^209^]

The failure of qcrCAB knockout experiments in mycobacteria attests to the vital role of cytochrome bc1 in their metabolism. Accordingly, the mycobacterial cytochrome bc1 is a promising therapeutic target, taking into consideration its unique structural features in reference to its mammalian equivalent. Telacebec and prevacid are top examples of mycobacterial cytochrome bc1 inhibitors.^[197]^

#### 3.3.5 Cytochrome bd Oxidase

**Figure 6:**
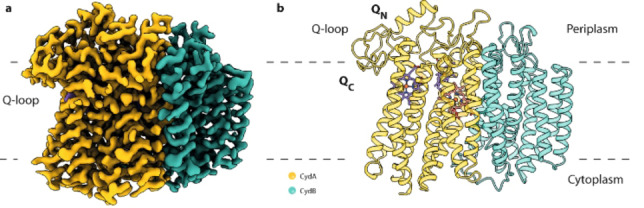
The structure of mycobacterial cytochrome bd oxidase and its spatial organization with respect to the mycobacterial cell membrane. (a) The dimeric enzyme structure visualized using a van der Waals surface representation. (b) A model of the enzyme complexed with its cofactors shown with respect to the cross‐section of the membrane (the membrane is represented as two horizontal lines with the lower facing the cytoplasm while the upper faces the periplasm). (Reproduced with permission from Ref. [24]**, ©** 2021 Creative Commons by 4.0).

The mycobacterial cytochrome bd oxidase consists of two subunits, CydA and CydB, embedded within the plasma membrane, and it is characterized by its pseudo‐symmetry. The two subunits of this oxidase are expressed by the cydABCD operon, restricted to only prokaryotic organisms. This oxidase functions secondary to the more energy‐efficient cytochrome bc1‐aa3 supercomplex in the oxidation of menaquinol and the concurrent production of metabolic water through the reduction of oxygen. Because of the higher oxygen affinity of cytochrome bd oxidase, compared to the supercomplex, the enzyme mainly serves the mycobacterial cell under hypoxic conditions, in addition to acting as a scavenger of reactive oxygen species. Therefore, the enzyme reinforces the mycobacterial survival and growth within the phagocytic cells of the host. Unlike the supercomplex, this oxidase does not translocate protons to the periplasmic space of the mycobacterial cell and thence has no contribution to the proton motive force. The catalytic subunit of this oxidase, CydA, possesses three heme groups, serving as an electron path between the substrate and the final electron acceptor, and a periplasm‐exposed Q‐loop domain with a highly specific binding site for menaquinol (Figure 6).[^23^, ^24^, ^195^, ^197^]

The knockout of the cydABCD operon does not alter mycobacterial growth under aerobic conditions, yet it augments the mycobacterial susceptibility to the inhibitors of their primary oxidase, cytochrome bc1‐aa3 supercomplex. Thus, the druggability of this secondary oxidase is only significant when combined with the simultaneous inhibition of the primary oxidase, which results in an accelerated eradication of the mycobacterial infection. No drugs have been issued for the mycobacterial cytochrome bd oxidase, and aurachin D is its only reported inhibitor.[^15^, ^195^, ^197^]

#### 3.3.6 ATP Synthase

**Figure 7:**
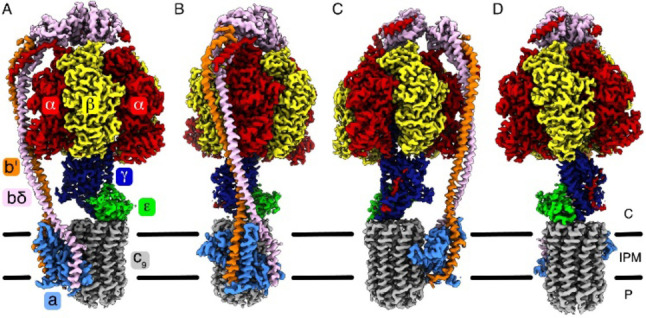
Four views of a cryo‐electron microscopy (EM) model of mycobacterial ATP synthase anchored, at the bottom of the figure, to the mycobacterial membrane represented by two broken horizontal lines. The standard annotations of the structural subunits of the enzyme are provided in panel (A). (Reproduced with permission from Ref. [211], © 2021 Creative Commons by 4.0).

The mycobacterial ATP synthase is a hetero 21‐mer structure, made of nine different subunits, architected into two functional domains, F_1_ and F_O_, in addition to a central stalk and a peripheral stalk (Figure 7). The 12‐mer F_O_ hydrophobic domain is inserted in the plasma membrane, while the hexamer F_1_ hydrophilic domain lies within the cytoplasm. The primary function of this moonlighting enzyme^[32]^ is harnessing the proton motive force generated by the electron transport chain in the manufacture of ATP. The shuttling of the high‐energy protons from the periplasmic space to the cytoplasm across the F_O_ domain of the ATP synthase triggers the rotary movement of its c‐ring, cylindrical nonamer of subunit c, traversing energy to the F_1_ domain which activates its catalytic subunits through conformational changes. The ATP production takes place at the catalytic site located at the interface between the activated, alternating alpha/beta subunits of the F_1_ domain. The beta subunit holds the binding regions for the ATP precursors, ADP and inorganic phosphate.[^16^, ^32^, ^195^, ^197^, ^210^, ^211^]

The mycobacterial ATP synthase is an established drug target for antimycobacterial compounds that are mainly used to treat resistant mycobacterial infections. Diarylquinolines and squaric acid derivatives are the most important classes of mycobacterial ATP synthase inhibitors, with bedaquiline as the most established, FDA‐approved drug belonging to diarylquinolines.[^29^, ^197^, ^212^, ^213^]

## 4 Molecular Modeling for an Efficient Discovery of Antimycobacterial Entities

Molecular modeling can provide insight into the selective targeting of mycobacteria (without harming human cells). When coupled with artificial intelligence (AI) algorithms, these *in silico* methods can narrow down potential antimycobacterial agents from an immense chemical space with efficiency and speed. Examples of such studies include:

- The discovery of three lead compounds against the polyketide synthase 13 thioesterase domain of Mtb (Mtb Pks13‐TE) through virtual screening of the Asinex antibacterial library.^[214]^
- The identification of novel hits against the decaprenylphosphoryl‐β‐D‐ribose‐2′‐epimerase (DprE1) of Mtb using an integrated computational approach to screen the ChEMBL database.^[215]^
- The design of Mtb Pks13‐TE hits using generative AI and molecular dynamics simulations.^[216]^

Nonetheless, antimicrobial resistance mechanisms (e. g. efflux pumps)^[217]^ and pharmacokinetic challenges (e. g. intracellular drug delivery)[^218^, ^219^, ^220^] present obstacles to efficacious and safe therapeutics for mycobacterial infections. More data is needed to complete the mapping of the resistomes of pathogenic and opportunistic mycobacteria.^[217]^ Additionally, the correlates of mycobacterial disease progression need further characterization to pinpoint the stages of the pathology, which is crucial for developing personalized therapeutic strategies.^[221]^ Nanotechnology and molecular informatics techniques can, together, enhance therapeutic breakthroughs, such as nano‐BacPROTACs.[^222^, ^223^]

## 5 Conclusions

Mycobacterial infections are historical and continue to present contemporary challenges to healthcare systems worldwide. The resistant nature of these opportunistic pathogens along with the economic burden of their diseases urge the discovery and development of innovative diagnostic and therapeutic tools. Targeting the energy metabolism of mycobacteria is an emerging therapeutic strategy, with several promising lead compounds and drug candidates progressing across the drug discovery and development pipeline. The oxidative phosphorylation pathway is crucial for the survival and proliferation of mycobacteria, illuminating a targetable vulnerability of these obligate aerobes. The recent advancements in the deciphering of the mycobacterial oxidative phosphorylation metabolic network together with the structural elucidation of many of its key players provide a ripe harvest of biological data awaiting its processing by computational biologists and bioinformaticians. The identification of potent and selective inhibitors of mycobacterial oxidative phosphorylation with a proper understanding of their structure‐activity relationships, combined with the rise of commercially available ultra‐large chemical libraries set the stage for cheminformaticians. This hybrid body of biochemical/pharmacological data altogether paves the road to computer‐aided drug discovery campaigns with the ultimate goal of finding valuable hits to join their predecessors in the pipeline. Further research is imperative to obtain a deeper comprehension of the mycobacterial host‐pathogen interactions, metabolic flexibility, and environmental reservoirs.

## 6 Lessons Learned and Outlook

1. Mycobacteria have plagued humans and other animals throughout history. They are opportunistic pathogens that are abundant in the environment, owing to their highly resilient physiology. Mycobacteria are nature‐refined disease agents, which serve to maintain the natural ecosystemic balance by obliterating vulnerable members within certain animal populations.
2. The epidemiology of mycobacterial diseases (e. g. TB) is both influenced by economic factors (such as poverty) and an influencer of global economies (e. g. epidemics in cattle).
3. Mycobacteria are ingenious in manipulating and hijacking phagocytic cells of immune systems. Hence, bactericidal therapeutic regimens are vital to avoid relapse.
4. It can be hypothesized that the global rise in pulmonary NTM infections (led by MAC pathogens) is promoted by the niche resulting from the global efforts to eradicate TB, in addition to the global increase in vulnerable populations who are susceptible to NTM infections (e. g. geriatric populations). Nevertheless, other environmental factors (e. g. pollution) must also be considered. Continuous epidemiological studies of mycobacterial infections are, thus, key to understanding the global infection dynamics of mycobacterial pathogens, and consequently predict/avoid future outbreaks of pulmonary mycobacterial infections.
5. Mycobacteria exploit sundry mechanisms to resist antimicrobials (e. g. efflux pumps, missense mutations in key amino acids within active sites of antimicrobial targets, and cell wall modifications) thereby evolving to superbugs, such as extensively drug‐resistant TB bacilli and the emerging pathogenic *M. abscessus* species. Innovative antimycobacterial agents are an unquestionable necessity to maintain our efficacious arsenal along this brutal evolutionary battle.
6. Mycobacteria are obligate aerobes, with varying hypoxia tolerance levels, equipped with powerful dormancy systems to persist respiratory challenges. The flexible respiratory metabolic network of mycobacteria entails a systematic approach to evaluating the druggability of the different mycobacterial respiratory enzymes.
7. Polymorphism of respiratory enzymes across the diverse mycobacterial species imposes significant challenges on the repositioning of antimycobacterial agents, targeting the mycobacterial respiratory machinery, against the multitude of pathogenic mycobacterial species. Further lead optimization after repositioning is, hence, necessary to ensure the efficacy of the repositioned antimycobacterial against the species in question.
8. The disparity of current literature on the respiratory machinery of NTM presents a pronounced hurdle to designing bactericidal agents targeting the NTM respiratory pathway.
9. In silico drug discovery techniques are feasible, powerful, and cost‐effective tools to study and extend the available data on mycobacterial respiratory machinery.
10. Further research is paramount to tackle the health challenges associated with the array of mycobacterial pathogens.

## Abbreviations

ADP: adenosine 5‘‐diphosphate
ATP: adenosine 5‘‐triphosphate
CD1: cluster of differentiation 1
COPD: chronic obstructive pulmonary disease
ENL: erythema nodosum leprosum
ETC: electron transport chain
FAD: flavin adenine dinucleotide
GM‐CSF: granulocyte‐macrophage colony‐stimulating factor
GPL: glycopeptolipid
HD: Hansen's disease
ICAM‐1: intercellular adhesion molecule 1
JAG1: jagged 1
MABC: *Mycobacterium abscessus* complex
MAC: *Mycobacterium avium* complex
MLC: *Mycobacterium leprae* complex
Mtb: *Mycobacterium tuberculosis*
M1: *pro*‐inflammatory macrophage
M2: *anti*‐inflammatory macrophage
MTC: *Mycobacterium tuberculosis* complex
NADH: nicotinamide adenine dinucleotide (reduced form)
NDH‐1: NADH dehydrogenase type I
NDH‐2: NADH dehydrogenase type II
NTM: nontuberculous mycobacteria
PI3P: phosphatidylinositol 3‐phosphate
PGL‐1: phenolic glycolipid‐1
PROTACs: Proteolysis targeting chimeras
PtpA: protein‐tyrosine phosphatase A
PtpB: protein‐tyrosine phosphatase B
PTX3: pentraxin‐3
SapM: secreted acid phosphatase M
SDH: succinate dehydrogenase
TB: tuberculosis
Th: T helper (cell)
TNF: tumor necrosis factor
VPS33B: vacuolar protein sorting 33B
V‐ATPase: vacuolar ATPase
WHO: World Health Organization
XDR: extensively drug‐resistant

## Glossary of Biomedical Terms

- **Apoptosis**: Programmed cell death, a regulated process by which cells undergo an orderly death to eliminate damaged or unnecessary cells.
- **ATP synthase**: An enzyme that catalyzes the synthesis of the energy storage molecule adenosine triphosphate (ATP) from adenosine diphosphate and inorganic phosphate by harnessing the proton gradient across the membrane associated with respiratory complexes.
- **Autophagy**: A cellular process where cells degrade and recycle their own components, often in response to stress or nutrient deprivation.
- **Axenic culture conditions**: Laboratory conditions where a single species of microorganisms is cultured without any contamination by other species.
- **Druggability**: The likelihood that a biological target can be effectively inhibited or activated by a drug.
- **Electron transport chain (ETC)**: A series of coupled exergonic redox reactions, mediated by protein complexes and electron shuttles occurring in a respiratory membrane, which transfer electrons eventually to the universal electron acceptor (triplet (respiratory) oxygen, O_2_, in most organisms) leading to the consequential production of ATP through harvesting the energy stored in the resulting proton gradient.
- **Etiologic Agents**: Microorganisms or pathogens that cause disease.
- **Ghon focus**: The initial area of infection in the lungs caused by *Mycobacterium tuberculosis*, often seen in primary tuberculosis.
- **Hansen's Disease**: The clinically‐precise name for leprosy, a chronic infectious disease caused by *Mycobacterium leprae* complex.
- **Hepatomegaly**: Enlargement of the liver.
- **Immunomodulation**: The alteration of the immune response, either by enhancing or suppressing it, often for therapeutic purposes.
- **Isoforms**: Different forms of the same protein that arise from gene splicing or other modifications.
- **Jagged 1**: A transmembrane protein involved in the Notch signaling pathway which is important in cellular development and cell fate determination.
- **Lymphadenopathy**: Disease or enlargement of the lymph nodes.
- **Molecular phylogeny**: The analysis of the genetic sequences of organisms to determine their evolutionary relationships.
- **Moonlighting enzyme**: An enzyme that performs multiple, often unrelated, functions beyond its primary catalytic activity.
- **Multibacillary**: Refers to an infection, especially leprosy, characterized by a large number of bacteria (bacilli).
- **Mycobacteria**: A genus of bacteria that includes pathogens known to cause serious diseases in mammals, such as tuberculosis and leprosy.
- ***Mycobacterium abscessus*** **complex (MABC)**: A group of rapidly growing mycobacteria associated with lung and skin infections, particularly in individuals with underlying conditions.
- ***Mycobacterium avium*** **complex (MAC)**: A group of bacteria that includes *Mycobacterium avium* and *Mycobacterium intracellulare*, which can cause lung disease, especially in immunocompromised individuals.
- ***Mycobacterium leprae*** **complex (MLC)**: The group of bacteria responsible for leprosy, primarily *Mycobacterium leprae*.
- ***Mycobacterium smegmatis***: A non‐pathogenic species of mycobacteria often used as a model organism in research.
- ***Mycobacterium tuberculosis*** **complex (MTC)**: A group of closely related mycobacterial species that cause tuberculosis, including *Mycobacterium tuberculosis*.
- **Nonchromogens**: Slow‐growing mycobacteria that do not produce pigment when grown in the dark.
- **Nontuberculous mycobacteria (NTM)**: Mycobacterial species other than *Mycobacterium tuberculosis* complex and *Mycobacterium leprae* complex that can cause pulmonary and other infections.
- **Obligate aerobes**: Organisms that require oxygen to grow.
- **Obligate pathogenic species**: Species that can only survive and propagate by causing disease in a host.
- **Operon**: A functioning unit of genomic DNA containing a cluster of genes under the control of a single promoter.
- **Opportunistic pathogens**: Organisms that cause disease primarily in individuals with weakened immune systems.
- **Oxidative phosphorylation**: The metabolic pathway where cells use enzymes to oxidize nutrients, thereby releasing energy used to form ATP by the intermediacy of the electron transport chain.
- **Paresthesia**: Abnormal sensations on the skin, such as tingling, pricking, or numbness.
- **Paucibacillary**: Refers to an infection, especially leprosy, characterized by a small number of bacteria (bacilli).
- **Phagocytic cells**: Cells that engulf and digest foreign particles, bacteria, and dying cells.
- **Phagolysosome**: A cellular structure formed by the fusion of a phagosome with a lysosome, where ingested material is digested.
- **Phagosome**: A vesicle formed around a particle engulfed by a phagocyte via phagocytosis.
- **Polymorphism of respiratory complexes**: The existence of multiple forms of respiratory complexes, contributing to variations in the electron transport chain and its associated energy transduction.
- **Pre‐Columbian Era**: The period in the Americas before the arrival of Christopher Columbus in 1492.
- **PROTACs (proteolysis targeting chimeras)**: Are a class of small molecules that target specific proteins for degradation by the cell's proteasome.
- **Proton‐motive force (PMF)**: The combined electrical and chemical potentials generated across a membrane such as the inner mitochondrial membrane as a by‐product of the electron transport chain, and which drives ATP synthesis.
- **Repositioning of antimycobacterial agents**: The strategy of using existing drugs approved for treating a specific mycobacterial infection to treat another (mycobacterial) disease.
- **Resistome**: The set of all the antibiotic resistance genes and their precursors in pathogenic or non‐pathogenic bacteria.
- **Respirasome**: A supercomplex of the electron transport chain that enhances the efficiency of electron transfer, and hence, the efficiency of ATP production.
- **Respiratory supercomplex**: An assembly of multiple respiratory chain complexes that function together to optimize electron transport within the electron transport chain.
- **Supercomplex**: A stable assembly of several enzyme complexes that work together to carry out a specific biological function.
- **Tuberculoid and lepromatous Hansen's disease**: The two main forms of leprosy, with tuberculoid being less severe and lepromatous being more severe and infectious.
- **Uncoupling agents**: Compounds that disrupt the proton gradient across the membrane associated with respiratory complexes *without resulting in ATP production* by “short‐circuiting” the oxidative phosphorylation and dissipating the proton motive force in the form of heat.
- **Ziehl‐Neelsen stain**: A staining technique used to detect acid‐fast bacteria, such as *Mycobacterium* species.

## Conflict of Interests

The authors declare no conflict of interest.

## Biographical Information

*Islam K. Matar. B.Pharm.Sci. (Misr University for Science and Technology), Postgraduate Diploma in Drug Discovery (Cairo University), is a M.Sc. Candidate in Applied Science at Saint Mary's University. His current research is focused on using molecular modelling/informatics for the discovery and development of therapeutic entities, with a current focus on antimicrobial agents*.

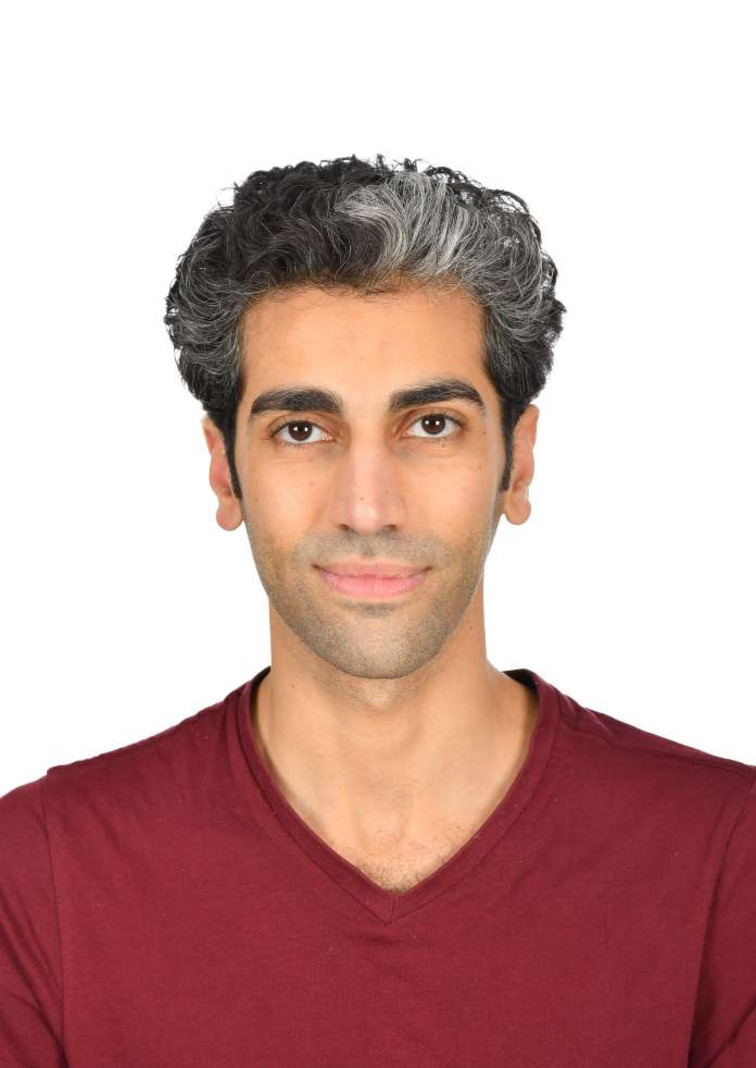

## Biographical Information

*Zhongmin Dong. Professor of Biology at Saint Mary's University, Canada. Previously has been a postdoctoral fellow at Queen's University, and Visiting Scientist at Leiden University, and the Australian National University. Works on plant‐microbe interactions have been a consistent theme, beginning with his PhD research project on a symbiosis of sugarcane with diazotrophic bacteria at Carleton University. His research set the foundation of the hydrogen fertilization theory that hydrogen gas released by legume nodules increases soil fertility*.

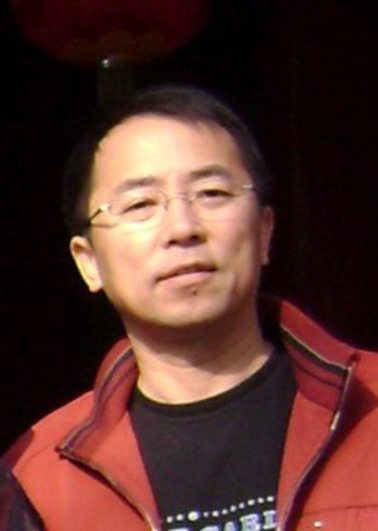

## Biographical Information

*Chérif F. Matta. B.Pharm.Sci. (Alexandria University), Graduate Diploma (Sadat Academy), PhD (McMaster University), HDR (Université de Lorraine), FRSA, FRSB, FInstP, FRSC, FAAS, FAAAS is Professor and Chair of the Dept. of Chemistry and Physics at Mount Saint Vincent University. Prof. Matta counts ca. 200 publications including 4 books and has given hundreds of invited and keynote lectures. His research focuses on theoretical and computational chemistry and mitochondrial biophysics. Matta has received several national and international awards for his work*.